# Correction: Explanation of the potential mechanism of *Sophora flavescens* Ait and processed products treating ulcerative colitis based on the analysis method of “chemical composition characterization-target prediction”

**DOI:** 10.1039/d6ra90043g

**Published:** 2026-04-30

**Authors:** Xiaoting Wang, Yue Liu, Tianni Jiang, Xiaodong Lv, Hui Gao

**Affiliations:** a Liaoning University of Traditional Chinese Medicine Shenyang Liaoning 110847 China deanoftcm@126.com; b School of Pharmacy, Liaoning University of Traditional Chinese Medicine Dalian 116600 China gaohuitcm@163.com; c Liaoning Agricultural Vocational and Technical College Yingkou 115009 China

## Abstract

Correction for ‘Explanation of the potential mechanism of *Sophora flavescens* Ait and processed products treating ulcerative colitis based on the analysis method of “chemical composition characterization-target prediction”’ by Xiaoting Wang *et al.*, *RSC Adv.*, 2025, **15**, 11354–11369, https://doi.org/10.1039/D4RA04760E.

The authors regret that an author was omitted from the authorship list of the manuscript. The corrected authorship is as shown above.

The Royal Society of Chemistry apologises for these errors and any consequent inconvenience to authors and readers.

